# Supplementary material for: Leaf Membrane Stability under High Temperatures as an Indicator of Heat Tolerance in Potatoes and Genome-Wide Association Studies to Understand the Underlying Genetics
Source: Plants (Basel). 2024 Aug 6;13(16):2175. doi: 10.3390/plants13162175 (PMC11359314; doi:10.3390/plants13162175)
Supplement: Supplementary file 1 [file plants-13-02175-s001.zip › Supplementary_Ifeduba_Electrolyte Leakage Assay_Submitted.pdf]

S1: Relative electrolyte conductivity (REC) of 215 potato clones evaluated at 50°C after four hours of heat stress using leaf tissues in a water bath. Each genotype was replicated three times, and five leaf discs of 5cm were collected per sample. The best linear unbiased estimator (BLUE) means were used, and the genotypes were organized according to market groups.

S/N	Genotypes	Market Group	REC	Mean Separation
1	AOTX96216-2Ru	Russet	99.59	A
2	COTX08121-4Ru	Russet	88.71	C-F
3	ATX15120-1Ru	Russet	86.94	D-H
4	AOTX95265-2ARu	Russet	86.35	F-K
5	AOTX96216-1Ru	Russet	84.48	G-P
6	ATX9202-3Ru	Russet	84.35	G-P
7	COTX08121-1Ru	Russet	84.20	H-R
8	ATX97147-4Ru	Russet	84.12	H-S
9	TX08350-12Ru	Russet	83.91	I-T
10	ATTX98468-5Ru/Y	Russet	83.81	J-U
11	COTX13029-2Ru/Y	Russet	82.63	M-Z
12	Russet Norkotah 112	Russet	81.19	S-G1
13	TX13590-9Ru	Russet	81.04	T-H1
14	AOTX95265-2Ru	Russet	79.94	Z-O1
15	AOTX96075-1Ru	Russet	79.90	Z-O1
16	Russet Burbank	Russet	79.82	Z-O1
17	ATX84706-2Ru	Russet	79.64	Z-P1
18	COTX05095-2Ru/Y	Russet	79.17	C1-Q1
19	TXA549-1Ru	Russet	79.03	D1-S1
20	COTX09052-1Ru	Russet	78.10	H1-V1
21	ATX84378-6Ru	Russet	77.72	K1-W1
22	MWTX2609-4Ru	Russet	77.70	K1-W1
23	COTX1723-2Ru	Russet	76.71	P1-Z1
24	COTX08063-2Ru	Russet	76.65	P1-Z1
25	COTX09052-2Ru	Russet	76.62	Q1-Z1
26	AOTX95265-4Ru	Russet	75.75	T1-C2
27	AOTX98202-1Ru	Russet	75.67	T1-C2
28	AOTX98152-3Ru	Russet	74.60	X1-G2
29	ATX9332-8Ru	Russet	74.18	Y1-I2
30	COTX10080-2Ru	Russet	74.09	Z1-J2
31	Russet Norkotah	Russet	72.75	C2-R2
32	NDTX1791-1Ru	Russet	72.60	D2-S2
33	TX61216-1Ru	Russet	72.49	D2-T2

34	Sierra Gold™	Russet	71.35	H2-X2
35	COTX09022-3RuRE/Y	Russet	70.79	M2-B3
36	AOTX98096-1Ru	Russet	70.74	N2-B3
37	AOTX95265-1Ru	Russet	70.73	N2-B3
38	MWTX548-2Ru	Russet	70.33	O2-C3
39	AOR07781-2	Russet	70.28	O2-D3
40	Russet Norkotah 102	Russet	70.13	Q2-E3
41	ATX9312-1Ru	Russet	70.10	Q2-E3
42	COTX04303-3Ru/Y	Russet	69.22	V2-H3
43	Russet Norkotah 296	Russet	68.87	W2-I3
44	POR06V12-3	Russet	68.63	X2-J3
45	AOTX98137-1Ru	Russet	68.17	Z2-L3
46	PA99N82-4	Russet	67.54	C3-M3
47	Russet Norkotah 278	Russet	67.33	C3-M3
48	TXNS249	Russet	67.29	D3-N3
49	AO7781-10LB	Russet	66.96	F3-O3
50	ATTX10007-1Ru	Russet	66.93	F3-O3
51	ATX99013-1Ru	Russet	66.47	G3-Q3
52	A02449-100LB	Russet	66.16	I3-S3
53	Reveille Russet	Russet	63.41	R3-Y3
54	ATX13287-1Ru	Russet	63.30	S3-Y3
55	Vanguard Russet	Russet	63.15	T3-Y3
56	Stampede Russet	Russet	62.05	V3-A4
57	Krantz	Russet	59.43	A4-D4
58	COTX13215-2Ru	Russet	57.99	B4-E4
59	ATX15097-1Ru	Russet	56.93	C4-E4
60	COTX08322-10Ru	Russet	56.79	D4-E4
61	Russet Norkotah 223	Russet	53.72	F4-G4
62	COTX17304-1Ru	Russet	47.00	I4
63	NDTX060700C-1W	Chip	94.47	B
64	COTX17288-3W	Chip	86.42	F-J
65	TX19456f-2W	Chip	85.48	G-M
66	AORTX09037-5W/Y	Chip	85.31	G-N
67	ATTX10333-1W/Y	Chip	84.62	G-O
68	NDTX071109C-1W	Chip	84.52	G-P
69	NDTX12471-6W/Y	Chip	84.49	G-P
70	TX1475-3W	Chip	84.36	G-Q
71	NDTX12203AB-1W	Chip	84.11	H-S

72	Atlantic	Chip	82.50	M-A1
73	TX17846-1W	Chip	81.25	R-G1
74	TX05249-11W	Chip	80.91	T-I1
75	NDTX1287B-1W	Chip	80.90	U-I1
76	ATTX95490-2W	Chip	80.63	W-K1
77	AORTX09037-1W/Y	Chip	80.21	Y-N1
78	COTX16013-5W	Chip	79.64	Z-P1
79	NDTX14263BC-3W	Chip	79.07	D-R1
80	NDTX081644CAB-2W	Chip	79.06	D-R1
81	TX09396-1W	Chip	78.84	D-S1
82	NDTX187Y-1W	Chip	78.28	G-U1
83	NDTX091908AB-2W	Chip	77.79	J-W1
84	TX12484-3WZC	Chip	77.75	K1-W1
85	NDTX081648CB-13W	Chip	76.11	R1-A2
86	COTX10097-2W	Chip	75.73	T1-C2
87	COTX03187f-1W	Chip	74.34	Y1-H2
88	NDTX081648CB-4W	Chip	74.30	Y1-H2
89	NDTX14247CAB-2W	Chip	73.79	Z1-M2
90	NDTX14247CAB-1W	Chip	73.17	A2-P2
91	TX05249-10W	Chip	72.48	D2-T2
92	AORTX11913-3W/Re	Chip	71.66	G2-W2
93	TX12484-2WZC	Chip	71.21	I2-Y2
94	A05214-3LB	Chip	70.87	L2-A3
95	AOTX95309-1W	Chip	69.97	R2-E3
96	NDTX1482YB-1W	Chip	69.26	V2-G3
97	TX11461-3W	Chip	66.68	F3-P3
98	TX12484-4W	Chip	66.22	H3-S3
99	NDTX14362AB-1W	Chip	65.47	K3-U3
100	NDTX1244-3W/Y	Chip	64.46	N3-W3
101	TX11461-2W	Chip	62.73	U3-Z3
102	NDTX081648CB-1W	Chip	61.37	X3-A4
103	NDTX059828-2W	Chip	56.66	D4-F4
104	TX17797s-11Y/Y	Yellow	94.72	B
105	Tacna	Yellow	90.08	C
106	Gold Nugget	Yellow	81.54	P-E1
107	Nautilus	Yellow	81.27	R-G1
108	Inka Gold	Yellow	80.77	V-J1
109	Tokio	Yellow	80.32	X-N1

110	COTX10226-1Wre/Y	Yellow	78.52	F1-T1
111	ATX11684f-2W/Y	Yellow	78.11	H1-V1
112	TX17805-10PYpinto/Y	Yellow	75.82	T1-B2
113	ATX05202s-3W/Y	Yellow	74.61	X1-G2
114	COTX03079-1W/Y	Yellow	73.84	Z1-L2
115	COTX05249s-3W/Y	Yellow	73.57	A2-N2
116	California Gold	Yellow	73.50	A2-N2
117	COTX10073s-1W	Yellow	72.31	E2-U2
118	Yukon Gold	Yellow	72.13	F2-V2
119	TXYG107	Yellow	72.12	F2-V2
120	TXYG55	Yellow	71.00	K2-A3
121	TX17763-2Y/Y	Yellow	69.57	T2-F3
122	NDTX081451CBs-1Y/Y	Yellow	69.13	V2-I3
123	TXYG105	Yellow	69.11	W2-I3
124	NDTX1246-5W/Y	Yellow	69.04	W2-I3
125	TXYG79	Yellow	67.86	B3-L3
126	Granola	Yellow	67.45	C3-N3
127	TX1673-1W/Y	Yellow	66.40	G3-R3
128	COTX10138s-7Wpe/Y	Yellow	66.26	G3-S3
129	White LaSoda	Yellow	63.73	P3-Y3
130	COTX10118-1Wre/Y	Yellow	59.58	A4-D4
131	TX17734-1Y/Y	Yellow	59.11	A4-D4
132	COTX10118-4Wpe/Y	Yellow	57.43	C4-E4
133	ATX13134-3W/Y	Yellow	50.11	H4
134	Dubloon	Yellow	48.94	H4-I4
135	ATX06264s-4R/Y	Red	89.62	C-E
136	ATTX98444s-16R/Y	Red	86.87	D-I
137	ATTX98465-1R/Y	Red	86.67	E-J
138	ATTX10265-4R/Y	Red	86.06	F-L
139	ATTX11633-1RYpinto/Y	Red	85.45	G-M
140	COTX15083-1R	Red	85.29	G-N
141	ATTX98462s-3R/Y	Red	84.68	G-O
142	COTX05211-4R	Red	84.67	G-O
143	TX17742-19R/R	Red	83.69	J-V
144	NDTX14156-2RWpinto/Y	Red	83.38	K-W
145	Waneta	Red	83.27	L-X
146	PORTX03PG25-2R/R	Red	82.44	N-B1
147	Sierra Rose™	Red	82.25	O-C1

148	COTX15111-1R	Red	81.80	O-C1
149	ATTX05175s-1R/Y	Red	81.45	Q-F1
150	COTX00104-6R	Red	81.08	T-H1
151	ATTX06246-1R	Red	80.56	W-L1
152	ATTX03516-2R	Red	80.35	X-N1
153	ATX11586-2R/Y	Red	80.20	Y-N1
154	COTX94216-1R	Red	80.01	Y-O1
155	TX11461-1R	Red	79.52	A1-Q1
156	NDTX059759-3R/Y Pinto	Red	79.45	B1-Q1
157	PTTX05PG07-1W	Red	79.38	C1-Q1
158	NDTX14156A-3RWpinto/Y	Red	78.95	D1-S1
159	ATX05186s-1R	Red	78.54	E1-T1
160	NDTX059761-1R/R	Red	77.67	K1-W1
161	Rio Rojo	Red	77.62	L1-W1
162	NDTX092237C-2R/R	Red	77.54	M1-X1
163	COTX05211-5R	Red	77.36	N1-X1
164	COTX02293-4R	Red	77.18	O1-Y1
165	NDTX5438-11R	Red	76.04	S1-B2
166	BTX2332-1R	Red	75.42	U1-D2
167	ATTX98453-11Br	Red	74.55	X1-G2
168	AOTX93483-1R	Red	73.91	Z1-K2
169	NDTX5067-2R	Red	73.59	A2-N2
170	TX17805-1RWpinto/R	Red	73.23	A2-O2
171	NDTX050184s-1R/Y	Red	73.04	B2-Q2
172	NDTX1753Y-1R	Red	72.77	C2-R2
173	Sarpo Mira	Red	72.67	D2-R2
174	COTX05211-7R	Red	72.58	D2-S2
175	TX14646-1RWpinto/Y	Red	70.19	P2-D3
176	NDTX4828-2R	Red	69.96	R2-E3
177	TX18192-2R/Y	Red	69.66	S2-F3
178	NDTX9-1068-11R	Red	68.65	X2-J3
179	ATTX98453-6R	Red	68.30	Y2-K3
180	NDTX731-1R	Red	68.04	A3-L3
181	TX17805-8R/R	Red	67.80	B3-L3
182	Red LaSoda	Red	67.14	E3-N3
183	TX17788f-4R/R	Red	66.89	F3-O3
184	NDTX071258Bs-1R	Red	66.80	F3-O3
185	ATTX98453-3R	Red	66.16	I3-S3

186	COTX02172-1R	Red	65.74	J3-T3
187	ATTX98466-5R/WR	Red	65.51	K3-U3
188	COTX94218-1R	Red	65.24	L3-U3
189	NDTX4271-5R	Red	64.07	O3-X3
190	Unica	Red	63.06	T3-Y3
191	NDTX4784-7R	Red	61.67	W3-A4
192	ATTX98448-6R/Y	Red	61.51	W3-A4
193	BTX2103-1R/Y	Red	61.35	X3-A4
194	ATTX01178-1R	Red	61.31	X3-A4
195	ATX02263-1R/Y	Red	60.73	Y3-B4
196	NDTX050169s-1R	Red	55.63	E4-G4
197	ATTX98493-2P/P	Purple	89.68	C-D
198	COTX10138-15Wpe/Y	Purple	87.31	C-G
199	COTX04050s-1P/P	Purple	82.97	M-Y
200	COTX08365f-3P/P	Purple	81.43	Q-F1
201	COTX16078-9PWpinto/Y	Purple	80.53	W-M1
202	COTX05082-2P/P	Purple	79.84	Z-O1
203	COTX17016f-2P/P	Purple	79.54	A1-Q1
204	NDTX14156-3P/WP	Purple	79.19	D1-Q1
205	NDTX092238Cs-1P/W	Purple	73.45	A2-N2
206	NDTX081618-1P/P	Purple	71.24	I2-Y2
207	ATTX88481-1P/W	Purple	71.12	J-Z2
208	ATTX99325-1P	Purple	70.69	N2-B3
209	ATTX98500-2P/Y	Purple	70.64	N2-B3
210	TX17742-5YPpinto/YP	Purple	70.13	Q2-E3
211	TX08325-2P/YP	Purple	69.47	U2-F3
212	CO112-F2-2P/P	Purple	64.70	M3-V3
213	TX17742-23P/P	Purple	64.00	O3-X3
214	TX12474-1P/R	Purple	53.50	G4
215	COTX08365-1P/P	Purple	49.88	H4-I4

Reference varieties: Heat tolerant – Vanguard Russet [63.15], Reveille Russet [63.41], Heat sensitive – Atlantic [82.50], Russet Burbank [79.82] and Intermediate genotype – Sierra Gold^{TM} [71.35]. Genotypes with REC value > 79% were color-coded blue and considered extreme ion leakage, while those with REC < 70% were considered low ion leakage and color-coded red. Others were considered intermediate (black).

P/Y = Purple, yellow flesh; P/P = Purple, purple flesh; R = Red, white flesh; R/R = Red, red flesh; R/Y = Red, yellow flesh; Ru = Russet, white flesh; Ru/Y = Russet, yellow flesh; RuRE/Y = Russet, red-eye yellow flesh; RYPinto/Y = Red, yellow pinto, yellow flesh; W = White, white flesh; W/Y = White, yellow flesh; Wpe/W = White, purple eye, white flesh; Wre/Y = White, red-eye, yellow flesh; Y = Yellow, white flesh; Y/Y = Yellow, yellow flesh (https://potato.tamu.edu/reports/).

S2: Overall differences between market groups based on relative electrolyte conductivity (REC) of the 215 clones evaluated at 50°C after four hours of heat stress in the water bath.

Market Group	REC[1]	Range
Chip (41)	76.07 A	56.66 - 94.47
Red (62)	74.43 AB	55.63 - 89.62
Russet (62)	73.43 BC	47.00 - 99.59
Purple (19)	72.33 BC	49.88 - 89.68
Yellow (31)	71.35 C	48.94 - 94.72
Market Classes		

[1]REC = Relative electrolyte conductivity

Values followed by the same letter in a column are not statistically different at $p \leq 0.001$.
